# Geo-mechanical and geo-morphology characterisation of the cap rocks in the Niger Delta for potential carbon capture and storage

**DOI:** 10.1016/j.heliyon.2024.e31206

**Published:** 2024-05-14

**Authors:** Itai Mutadza, Sunday Sunday Ikiensikimama, Ogbonna Friday Joel

**Affiliations:** University of Port Harcourt, East-West Road, Port Harcourt, Nigeria

**Keywords:** Carbon sequestration, Cap rocks, Mineralogical, Geo-mechanical

## Abstract

The world has been battling climate change which is caused by an excessive amount of carbon dioxide in the atmosphere. Carbon sequestration in geological sinks was identified as one of the major methods to curb and reduce the amount of carbon dioxide in the atmosphere. The carbon dioxide stored in the geological formations must not escape back into the atmosphere. Characterisation of potential reservoirs for geological sequestration is pertinent for injectivity, capacity, and most importantly containment of carbon dioxide. The cap rock is one of the most important factors determining carbon sequestration success. This study focuses on the preliminary characterisation of the caprocks for evaluating potential subsurface storage of carbon dioxide based on their petrophysical characteristics. X-ray Diffraction and X-ray Fluorescence provided the mineralogical composition and geochemical data of the cap rocks while Scanning Electron Microscopy-EDS was used to identify the qualitative information on the micromorphological textural structure of the cap rocks in the Niger Delta. Conventional Triaxial experiments were used to determine the elastic and geo-mechanical strength of the caprocks. It was identified that the grain skeleton of caprocks in the region is predominated by Quartz, Albite, and Muscovite. The ratio of the tectosilicate and phyllosilicate minerals indicates that the cap rocks are silicate shale. The peak strength of the caprocks ranged from 48.85 to 80.50 MPa and are classified as strong rocks. The cap rocks showed a quasi-elastic behaviour after being subjected to compressive axial force. The elastic modulus of the caprocks was also observed. The characteristics exhibited by the shale caprock at the rock matrix level are highly favourable for sealing carbon dioxide and hence indicate capability for use in carbon sequestration.

## 1 Introduction

**Fig. 1:**
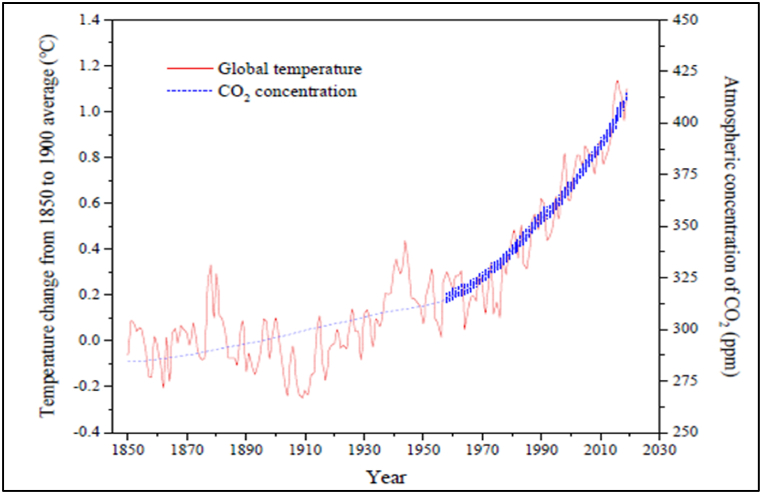
Correlation between the concentration of carbon dioxide and average global temperature increase [13].

An excessive amount of carbon dioxide has been expended in the atmosphere leading to an increase in the average global temperature [[1], [2], [3], [4]]. The carbon dioxide concentration has significantly increased from the preindustrial era from as low as 200 parts per million (PPM) to presently over 415 parts per million (PPM) under large-scale emissions [5,6]. It is estimated that over 24 gigatons (Gt) of carbon dioxide are produced annually from the use of fossil fuels, causing the atmospheric carbon dioxide concentration to significantly increase during the past century [7]. Hameli et al. [8] and Umar et al. [6] attested that carbon dioxide accounts for about 65 % of the greenhouse effect. This leaves carbon dioxide as the major contributor to global warming [9]. The increase in average temperature globally can be correlated to the increase in carbon dioxide concentration in the atmosphere [[10], [11], [12]]. Cao et al. [13], illustrated the correlation (see Fig. 1). The rise in global average temperature has in turn caused catastrophic events globally.

Efforts to curb the average temperature increase to 1.5^o^C was agreed upon in Paris during the Conference of Parties 21 (COP 21) [13]. However, according to various estimates of carbon dioxide emissions per year, the average world temperature is expected to rise by up to 4 °C over the next 50 years [14]. Carbon dioxide emissions have continued in large amounts, especially from burning fossil fuels and according to Hameli et al. [8], year 2021 recorded the all-time highest carbon dioxide emissions.

Carbon Capture and Storage in geological sinks has been identified as one of the possible significant ways to reduce the anthropogenic carbon dioxide in the atmosphere [[15], [16], [17], [18], [19]]. Sedimentary basins around the world have been identified as potential for carbon sequestration [4,9]. Espinoza et al. [20] suggests that sedimentary formations were chosen because it has been proven that natural carbon dioxide accumulates in them where pore pressure varies from hydrostatic pressure to lithostatic pressure, however, it is essential that the chosen basins be characterized. To seal off the carbon dioxide, a sealing caprock is essential [21,22]. The caprock sealing the reservoir formation also needs to be characterized to ascertain its integrity. Carbon dioxide injection may cause heat and pressure strains in the injection wellbore, the storage reservoir, and the caprock-reservoir interface, which can undermine the reservoir's integrity [23]. The carbon injection itself at high pressure can surpass pore pressure which may initiate fracture pathways resulting in compromised integrity of the sealing caprock [12]. Other processes such as drilling and completing wells through shale and mudstone strata require extra care because rock failure and deformation caused by wellbore instability can damage wellbores, resulting in increased permeability and possible leakage routes [4].

**Fig. 2:**
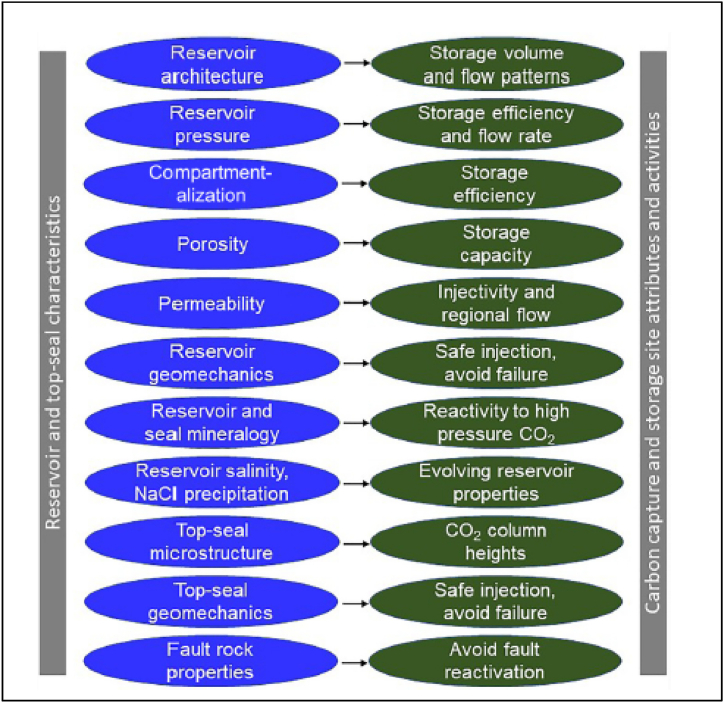
Caprock Characterisation attributes [25].

Dewhurst et al. [17] suggests that the cap rocks need to be characterised in terms of the mechanical properties-integrity, thickness and seal capacity whilst Raji et al. [24], added the assessment of the risk of leakage to the important factors. The geo-mechanical characterisation of carbon capture and storage formations is critical to ensuring that injection rates are as high as feasible to obtain acceptable efficiency while not being too high to risk reservoir integrity (fluid-fracturing). Worden [25], summarised the important characterisation attributes as shown in Fig. 2. These factors are influenced by the geological and petrophysical properties of the target formation [8]. The thrust of this research is to study the geological and petrophysical properties of cap rocks in the Niger Delta.

Unmineable coal seams, aquifers, oceans, salt caverns, mature oil fields and depleted oil and gas reservoirs are among the sedimentary basins that are used for carbon capture and storage [9,13,16,23]. There are certain precautionary characteristics that these geological basins should possess. These include a depth of 800 m, adequate storage, porous media, stable mineralogy and most importantly a sealing cap rock for containment of carbon dioxide [16,26,27]. The sealing caprock is crucial in ensuring that the injected gases are permanently confined, avoiding conductive fissures that would allow the gas to migrate to the surface and contaminate human-consumption water sources [14]. Nigeria has a potential for carbon sequestration, the most prevalent option being depleted oil and gas reservoirs, however, to the best of our knowledge none has been utilised for carbon sequestration. It is important to set geological sinks in Nigeria as it ranks second in the world in gas flaring [7,28]. Ojo & Tse [29] affirmed potential carbon storage in the Niger Delta reservoirs.

The depleted oil and gas reservoirs have a previously proven capacity to hold hydrocarbons [30]. The principle of operation predicts that carbon dioxide will occupy the pore spaces that were previously occupied by hydrocarbons [31,32]. Despite the proven capacity, there is a need to re-characterise these depleted oil and gas reservoirs to ensure successful sequestration [33]. The analogy of the occupied hydrocarbons gives some confidence in the cap rock's ability to impede carbon dioxide from moving back into the atmosphere or other overlying lithologies [26]. The Weyburn field project in Canada is one of the benchmarks that have been used to prove successful sequestration in oil field reservoirs [28], however, the carbon capture technology has never been practised in Nigeria where depleted and oil field reservoirs are common. The other advantage of using depleted oil and gas reservoirs is that there is already installed infrastructure that may be utilised for carbon sequestration with little or without alterations [26]. Saraf & Bera [34] puts it as an all-time geological sequestration with reduced expenses.

The petrophysical and petrographic properties of the depleted reservoirs are essential for the feasibility of carbon capture and storage [16]. To complement the petrophysical and petrographic properties, the mechanical strength of the cap rock must also be considered. Hangx et al. [23], found that the integrity of the caprock is dependent on the stress-strain changes that are exposed to the rock. According to Aminu et al. [26], and Gravogl et al. [35], the viable candidate technology for carbon dioxide subsurface storage should provide a minimum residence length of 1000 years and a leakage rate of less than 0.1 % annually. Injection and storage of carbon dioxide may drastically influence the geo-mechanical and geochemical characteristics of caprocks. The injection influence may make it easy for carbon dioxide to leak through the caprock due to its propensity to increase fluid pressure, lower effective stress and so weaken the rock. Loizzo et al. [31], suggested that depleted gas reservoirs have caprocks that have proven to seal pressurised fluids. It is however, still imperative to conduct characterisation to reduce the risk of the fluids particularly carbon dioxide from escaping back into the atmosphere.

Xie et al. [36], also suggested that the mechanical integrity of rocks is affected by the load and pressure induced on them. Zhang [37], recorded that the concern with depleted oil and gas reservoirs is that stress change induces compaction of the rocks which may lead to cemented matrix cracking and propagating the existent fissures more. The injectivity of carbon dioxide is dependent on the elastic and inelastic geo-mechanical attributes of the caprock [25]. The injection of carbon dioxide has the potential to induce seismic events that originate from the expulsion of stored energy caused by an increase in fluid pressure [4]. The induced seismic events may cause faulting and or fault reactivation. According to Edvardsen et al. [21], the strength of rocks and other porous materials is degraded when effective stresses surpass the material's intrinsic resistance, whether in tension or compression. With these narratives, the integrity of the silicate shale caprocks must be studied to ensure that the caprocks will not fail and release carbon dioxide. Huang et al. [38], indicated that the mechanical behaviour of rocks is essential for the success of carbon capture and storage. The In Salah (Algeria) carbon capture and storage project was halted in 2011 after the caprock started failing because of the injectivity pressure [26]. Worden [25], suggests that induced fracturing may be beneficial in the case of the reservoir rock itself as it enhances permeability however in the case of the sealing caprock it is a drawback.

Many studies have been done to ascertain the integrity of the reservoirs, a few have focused on the caprock. Previous work on the Niger Delta's potential for carbon capture and storage was carried out by Yahaya-Shiru et al. [28] and Ojo & Tse [29]. They focused on storage capacity using wireline logs and petrophysical properties such as permeability and porosity of the formations however they did not consider the mineralogical composition and geo-mechanical strength of these reservoirs and sealing caprocks. They limited their research to Swamp DepoBelt 1. The Niger Delta region is mainly dominated by shale cap rock which is well known for low permeability and low porosity which is a good characteristic to seal off carbon dioxide. Mineralogical determination is essential when considering carbon storage as it guides towards predicting possible geological reactions which in turn may possibly affect the integrity of the reservoir and the caprocks [39]. Worden [25] also highlighted the importance of considering geo-mechanical properties of caprocks to determine if they fracture after fluid pressure increases. Very little information is available on the mechanical integrity of the shale cap rocks in the Niger Delta.

In this study, we focus on the preliminary characterisation of the caprock in the Niger Delta for potential carbon sequestration covering the Deep Offshore (DO), Upper Delta (UD) and the Delta(D) depo-belts. The study focuses on mineral phases and triaxial geo-mechanical strength of the formations which were not considered in previous researches. The results obtained may be used as feed data for (a) modelling to investigate the impact of injection on a prospective carbon capture and storage (CCS) site, (b) used in the engineering calculation of injection pressure at the wellbore and (c) used to predict the potential reactions on the sealing caprock systems. Field assessment and in situ testing are beyond the scope of this study.

## 2 Geological setting

**Fig. 3:**
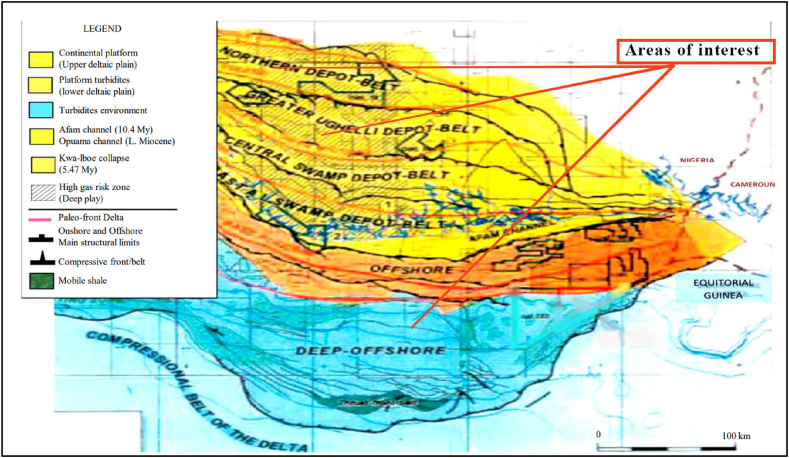
The deposit belts in the Niger Delta (modified by author) [42].

According to Yahaya-Shiru et al. [28] and Ojo & Tse [29], the favourable region for a significant potential carbon sequestration in Nigeria is the Niger Delta. The Niger Delta is located in the Southern part of Nigeria and it overlies over 256 000 km^2^ [40]. Ubani et al. [41], describes the location as the Gulf of Guinea in a rift triple junction which relates to the opening of the southern Atlantic which started in the later Jurassic to the Cretaceous. The Delta consists of 6 tide-influenced deposit belts as shown in Fig. 3. The Depo belts represent the successive growth of the delta. The Niger Delta consists of three lithological subsurface formations; the marine Akata Shales, the paralic Agbada Formation, and the continental Benin Formation [41]. Stratigraphically the Niger Delta consists of sedimentary rocks which range from sandstones and mudrock seal facies to shale rocks. The cap rock samples used in this research were obtained from Upper Delta (UD), Delta (D) and Deep Offshore (DO) belts. The interval of interest was approximately 3000 m–4000 m depth, with the samples from Upper Delta at 3 048 m, Deep Offshore at 3353 m and Delta at 3658 m.

## 3 Materials and methods

**Fig. 4:**
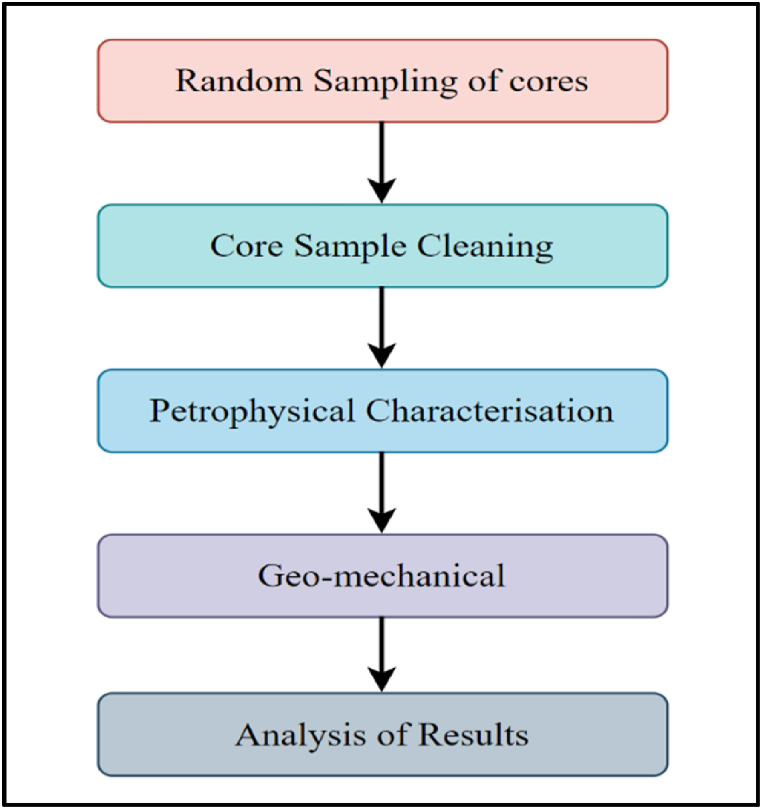
Sequential flow of the methodology

This research was carried out using cylindrical core samples that were randomly selected from three of the six depo belts in the Niger Delta. Core samples are extremely useful resources for better understanding the lithology of prospective carbon capture and storage reservoir formations and the sealing caprocks [25]. The cylindrical core samples with an average range diameter of 3.8–3.9 cm and length of 6.9–7.1 cm were drilled from the available core material. 12 samples were used in this study and 6 sample results which were reported were void of fractures. It is anticipated that the strength and elastic characteristics of these core samples are highly anisotropic. Mineralogical composition, micromorphological data, and geo-mechanical strength were determined in this study. X-Ray Diffraction (XRD), X-ray Fluorescence (XRF) and scanning electron microscopy with energy dispersive spectrum (SEM-EDS) housed at Spectral laboratory, Nigeria were used. Conventional Triaxial equipment used in this study is housed at the TU Delft University, the Netherlands. The Triaxial equipment was used to determine the geo-mechanical properties of the silicate shaly caprock. The flow of the methodology is shown in Fig. 4.

### 3.1 Procedure determination of mineralogical and chemical composition

The mineralogical petrographic characterisation X-ray Diffraction (XRD) analysis was carried out by Empyrean Malvern Panalytical Diffractometer whilst the chemical analysis was determined by X-ray fluorescent (XRF) Nitron 3000. For both the XRD and XRF analysis the cap rock samples were scraped to remove particles from the main cores. The samples were obtained from approximately 5 mm beneath the surface to avoid interference from particles that were in contact with the drilling fluids. The removed particles were ground further to produce fine particles less than 2 μm in size, which were then finely mixed and homogenised.

The homogenised powder sample was then prepared using the sample preparation block, placed in a borosilicate-glass sample holder was placed in the X-ray Diffraction meter. The samples were analysed using a reflection-transmission spinner stage with Theta-Theta settings. The initial analysis point for the 2*theta was 4°, and the 2*theta step was 0.026261 at 8.67 s each step. The Gonio Scan was utilised in combination with a 5 mm Width Mask. The intensity of diffracted X-rays was continually recorded while the sample and detector rotated through their respective orientations. When the material includes lattice planes with d-spacings adequate for diffracting X-rays at that value of, an intensity peak arises. Although each peak is made up of two distinct reflections (Kα1 and Kα2), for low 2*theta values, the peak positions overlap, with Kα2 appearing as a hump on the side of Kα1. Higher values of θ cause more separation. Typically, these merged peaks are classified as one. The 2λ location of the diffraction peak is commonly measured as the peak's centre at 80 % peak height. The XRD diffractograms were obtained using 45 kV voltage and a current of 40 mA with temperature regulated at 23^0^ C. The method is congruent to the one by El Bamiki et al. [43].

### 3.2 Petrographic analysis

To analyse the morphological structure and texture of the sampled rocks was viewed under a Phenom Prox model, Scanning Electron Microscope manufactured by Phenom World Eindhoven, Netherlands equipped with energy dispersive X-ray spectroscopy. The SEM imaging analyses were done at different scales and were collected at 15 KV.

### 3.3 Geo-mechanical strength

Dry triaxial experiments were carried out to ascertain the dry failure and dilation envelopes of the shale caprocks. The experiments were carried out at room temperature and a step wise confining pressure of 5 MPa, 7.5 MPa and a third higher pressure to simulate field conditions. Confining pressure is depth dependent. Before experimenting, the core samples were enveloped by a silicone resin layer to match the dimensions of the Hoekcell. The surfaces of the core samples were evened out to enable an equal distribution of the load. The core samples were put into the Hoekcell after being equipped with the exterior silicone resin layer. The material was subjected to hydrostatic pressure in the Hoekcell. (For procedural reasons, the axial pressure was kept slightly greater than the radial pressure). The radial pressure was held constant after a confining pressure of 5 MPa was established.

The axial load was then increased by loading the machine at 0.0005 mm/s deformation speed. After determining the commencement of yield, the sample was partially unloaded to establish a higher hydrostatic pressure of 7.5 MPa. The sample was loaded at a steady speed from the hydrostatic state till yield was noticed. The sample was slightly unloaded at this stage to prepare for the next hydrostatic pressure. Sample deformation (strain) was detected using a mix of strain gauges mounted directly to the samples and a linear variable displacement transducer (LVDT) measured the displacement. The sample was then loaded until total sample failure occurred under the final pressure regime. The methodology is comparable to the one by Hangx et al. [23]. The Young's Modulus of the individual caprocks was determined from the stress-strain graphs using the linear proportion of the curves. The accuracy of the Young Modulus was determined by the closeness of the R^2^ value being equal to 1.

## 4 Results and discussion

The bulk fraction's XRD analysis examination revealed the mineralogical assemblage and related relative abundances of minerals in the core samples by weight percentage (see Table 1). The grain skeleton of the caprocks in the Niger Delta are dominated by Quartz, Orthoclase and Albite. The trace elements are Garnet and Illite. Kaolinite was only found in the bottom cap rock of the Upper Delta (UDCB) region and the upper caprock of the Deep Offshore (DOCT) depo belt is devoid of Garnet see Table 1. The ratio of the tectosilicate and phyllosilicate minerals indicate that the cap rock in all three regions is silicate shale as suggested by Edvardsen et al. [21], Ubani et al. [41] and Du et al. [44]. The silicate shale samples have the requisite mineralogy and characteristic low permeability and porosity to serve as cap rocks for the depleted reservoirs. An ideal sealing caprock would be a non-reactive caprock, however, the findings of this study show reactive mineral composition in the caprocks. Despite containing some high end reactive clay minerals such as Chlorite and Muscovite the reactions are likely to be very low due to characteristic low permeability attributes of caprocks as attested by Edvardsen et al. [21]. The low permeability prevents the buoyant carbon dioxide plume from migrating upward.

**Table 1:** XRD Caprock Mineralogical Composition by weight %.

Mineral name	UDCT	UDCB	DOCT	DOCB	DCT	DCB
**Quartz**	55	51	59	49	56	53
**Illite**	2.3	8	11	0.1	0.9	1.6
**Chlorite**	5.7	1.4	8	8.1	1.5	3.3
**Orthoclase**	27	2.5	5.9	15.1	17	29
**Muscovite**	0.8	4	5	22	0.1	2.35
**Albite**	6	23	11	2.7	22	8.2
**Garnet**	3.4	4.3	0	3.2	1.9	2.14
**Kaolinite**	0	6	0	0	0	0

**Fig. 5:**
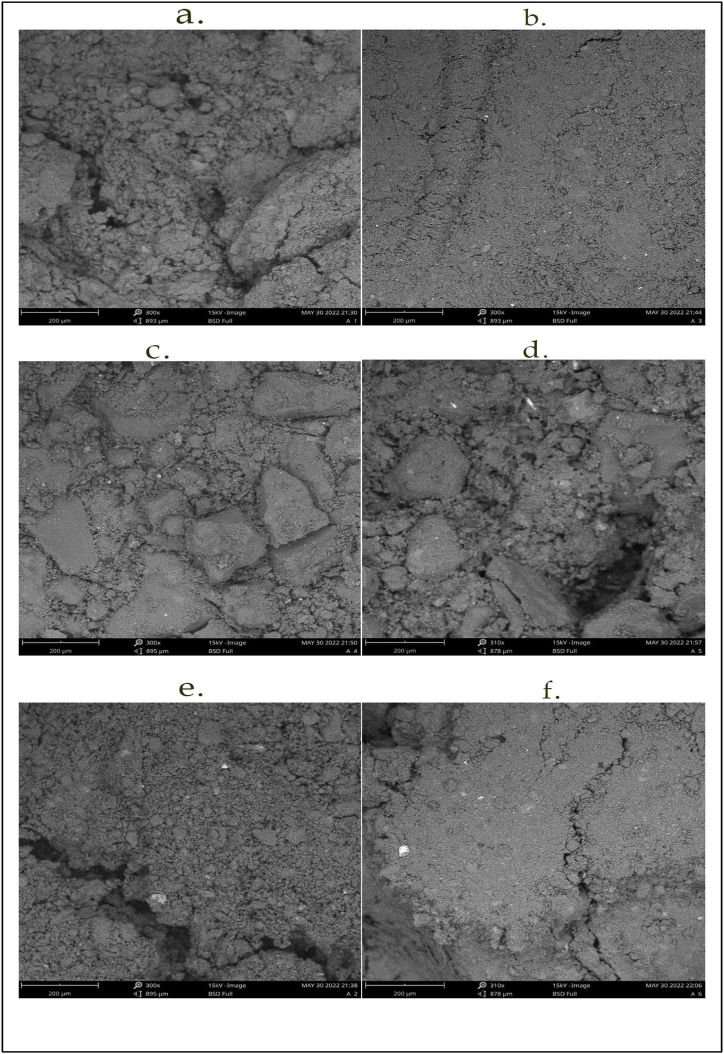
Micro structure of the samples taken by SEM-EDS a) UDCT b) UDCB c) DCT d) DCB e) DOCT f) DOCB

Following the petrographic examination by SEM (see Fig. 5(a–f).), the morphological structure of the sampled cores can be described as close-packed granular. The pore microstructure network was also observed. Upper Delta bottom caprock (UDCB) (see Fig. 5b.) and Deep Offshore top caprock (DOCT) (see Fig. 5e.) contained finely grained grains whilst Delta top caprock (DCT) and Delta bottom caprock (DCB) (see Fig. 5c and d) have uneven grains. The images indicate that the formations in the Upper Delta (UD) and Deep Offshore (DO) regions are skewed towards heterogeneous formations due to the mixture of the medium to fine grained rock particles whilst the Delta region is more homogeneous. Given the proportion of the minerals there is a high possibility of the cap rock trapping carbon dioxide stratigraphically or by residual trapping. This makes Niger Delta caprocks attractive candidates as sealing rocks for carbon dioxide storage.

Due to stratigraphic trapping, a plume of carbon dioxide is bound to concentrate at the reservoir-caprock interface and it is likely to react with the cap rocks which may alter the composition matrix of the caprock. The anticipated consequences are difficult to predict, however, they might result in either degradation or enhancement of caprock qualities. The preliminary mineralogical composition that was obtained in the Niger Delta was used to predict possible reactions with the injected carbon dioxide and other formation fluids. According to numerous pieces of literature, quartz is a strong mineral less susceptible to carbon dioxide. This is due to lack of metal ions in quartz as attested by Liu, [30]. However, other minerals like orthoclase, muscovite and chlorite are prone to dissolution and precipitation [16]. When these minerals come into contact with water, they release ions that migrate to the bulk solution through electrical interaction and random vibration inside the Helmholtz layer. These ions undergo the most significant potential shift across the Helmholtz layer, eventually forming activated complexes and new secondary minerals in the bulk solution.

All three contain a considerable amount of quartz; Upper Delta (UD) has an average of 53 %, (Deep Offshore) DO 54 % and Delta (D) 54.5 % by weight. Due to grain-to-grain contact, which is sufficiently quartz-cemented as shown by the SEM images in Fig. 5(a–f), the caprocks from all three depo belts are highly unlikely to be impacted by carbon dioxide reactions. A sufficient quartz grain to grain resistance on reactions was attested by Huang et al. [38]. Quartz is quite resistant to the acidity that is induced during carbon dioxide injection and also has a high frictional strength (frictional coefficient μ of about 0.85) stated by Bakker et al. [45]. The caprocks from all three sampled depo belts have the potential to seal carbon dioxide. This is attested by Chenrai [9] who highlighted that quartz is geometrically conformable to inversion seals.

Illite is another mineral that is less susceptible to acidity and our results show that the top cap rock in the Deep Offshore is the most less susceptible with 11 %, followed by Upper Delta with 2.3 % and lastly Delta with 0.9 % (see Table 1). The availability of this mineral in the top cap rock reduces the risk of carbon dioxide escaping back into the atmosphere. Another mineral, Chlorite, a Fe and Mg-rich mineral is highly likely to dissolve in mildly acidic formation water [25]. If there are any significant reactions to occur, the deep Offshore region is highly likely to be affected by the dissolution of chlorite followed by the Upper Delta and Delta is less likely to be affected as it contains less Chlorite. The reactions have been found to largely alter the reservoir by increasing porosity and permeability characteristics of the caprock and are likely to decrease rock strength hence the need to assess the extent of alteration. The extent of alteration may affect the integrity of the sealing caprock or the injectivity rates at which the fluids are pumped in this case carbon dioxide-brine solution. The reactions either enhance or degrade the cap rock strength depending on the ratios of the minerals that dissolve and those that precipitate. Orthoclase, Chlorite and Muscovite are principally dissolute. From the results shown in Table 1., the Upper Delta and Delta region will likely be affected by dissolution. However, the Upper Delta and Deep Offshore caprocks are highly likely to be of better quality than the Delta caprock as caprock quality correlates with Illite content. Illite is principally immune to high carbon dioxide partial pressures [33]. In addition to the suitable mineralogical composition the porosity and permeability of the reservoirs in the three depo-belts were found to be above the critical threshold ranges. The average porosity for the Delta dep-belt was 23.94 %, 11.22 % for Deep Offshore depo-belt and 11.72 % for the Upper Delta at standard atmosphere. The threshold porosity was attested to be 10 % by Eigbe [46]. The average permeabilities for Delta, Deep Offshore and Upper Delta at standard atmosphere were 183.5mD, 276mD and 279mD respectively. The threshold values for permeability were 150mD as attested by Eigbe [46] and Redondo [16].

**Fig. 6:**
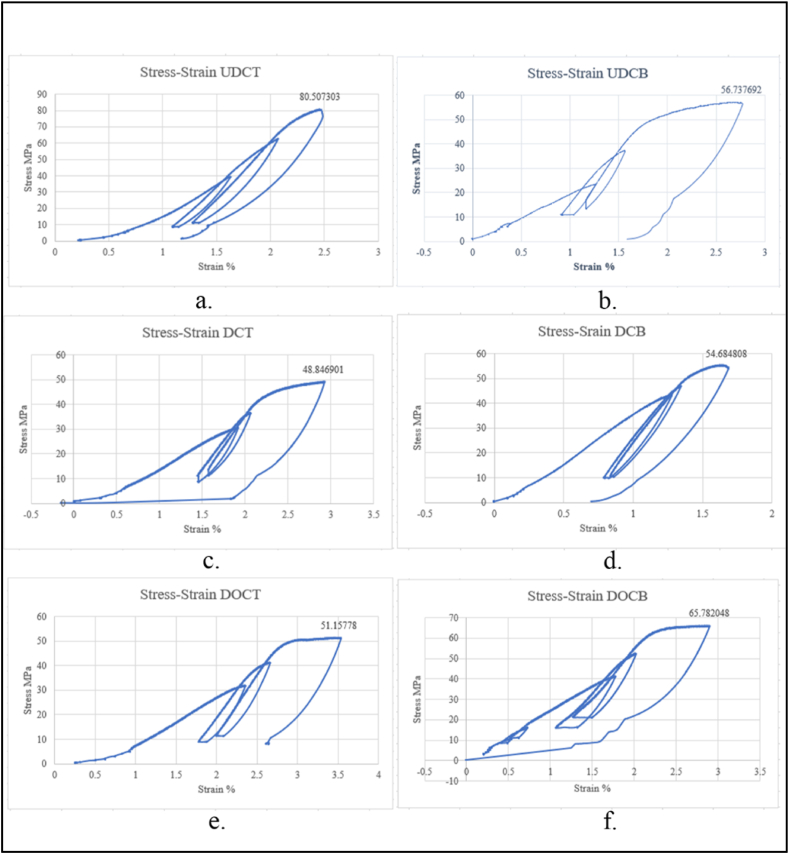
Stress-strain curves of a) UDCT, b) UDCB, c) DCT, d) DCB, e) DOCT, f) DOCB

We also studied the mechanical characteristics of the reported silicate shale sealing caprock system to find the optimum cap rock formation in the Niger Delta to operate as an effective and safe carbon dioxide seal. Our findings were not affected by pore pressure since the tests were carried out on dry samples hence insignificant pore pressure. All results were reported based on the Terzagh effective stress principle. To illustrate the normal real-time behaviour observed in the triaxial tests at room temperature with a step wise confining pressure of 5 MPa, 7.5 MPa and a third higher pressure the stress-strain curves of the caprocks in the Delta Region, Upper Delta and Deep Offshore are shown in Fig. 6(a–f). The rock core samples were subjected to three cycles of load and were run to fail. As shown in the axial stress versus strain graphs (Fig. 6 a-f), the cap rocks exhibited quasi-elastic behaviour before yielding. Similar observation of quasi-elastic was reported by Peter et al. [47] on porous rocks. The rocks subsequently failed at peak stress. The graphs show some fairly linear sections which indicate poro-elastic meaning the rocks are purely elastic at those sections. The initial non-linear sections before the poro-elastic section are attributed to the closure of pores and minor cracks within the rock samples. The non-linear sections after the poro-elastic section (before the peak stress) are attributed to the beginning of failure of the rock and starting of new microcracks. This behaviour is important when considering carbon storage as it determines the safety of the project. It is safer to operate below the yielding points of these rocks to avoid failure and escape of carbon into other lithologies and back into the atmosphere. It is hypothesised that mechanical behaviour is intimately connected to the mineralogical characteristics of the material however our results correlate with results from Hangx et al. [15] which show a poor correlation between strength and mineralogy.

By analysing the strengths of the caprocks it was observed that the compressive peak strength of the top Upper Delta caprock is 80 MPa whilst the compressive peak strength of the bottom caprock is around 56 MPa. The Deep Offshore had compressive peak strengths of 50 MPa with an axial strain of 3.5 % and 65 MPa with an axial strain of about 2.8 % for the top and bottom cap respectively whilst the Delta depo belt has compressive peak strengths of 60 MPa for the top caprock with an axial strain of about 2.9 % and 55 MPa for the bottom caprock with a strain of about 1.6 % (See Fig. 6(a–f)). The peak compressive strength results are congruent and even surpass 48–52 MPa which was reported by Rathnaweera [48]. Carey [49] reported a peak strength of 85 MPa for the shale cap rock they studied. The peak strengths are also in the ranges that were reported by Vallejo and Ferrer [50] for the shale and sandstones. With our findings the silicate shale caprocks can be classified as high strength rocks following the classification by Vallejo and Ferrer [50] Arsyad et al. [51] who classified 5–25 MPa as low strength, 25–50 MPa as medium strength and 50–100 MPa as high strength rocks. The experimental findings indicated an increase in strength with an increase in the confining pressure. The differences in the peak strengths at the same confining pressure could be attributed to differences in pore sizes of the different porous core samples, structure and pore connectivity (see Fig. 6.). According to Ye et al. [52], peak strength of rocks decreases with increase in pore size. Huang et al. [38], also had similar results. The graphs illustrate an increase in stiffness even though the samples are supposedly deforming at pore and grain scale due to the compressions. Stiffness being the ratio of applied load to vertical displacement.

The Young modulus of the caprocks in the Niger Delta was determined from the stress-strain relationship and they ranged from 18.54 to 39.775 MPa (see Table 2). The Young Modulus of the caprocks obtained in this research are congruent to the Young Modulus values by Hangx et al. [23]. In addition, the maximum differential stress (δ_1_ -δ_3_) max of the caprock rocks was determined, where δ_3_ = P_c_, P_c_ is confining pressure. The differential stresses ranged from 38.846901 to 70.507303 MPa (Highest P_c_ was taken to be 10 MPa) (see Table 2). The strength results of the cap rocks can be used to determine the maximum allowable injection pressure into the Niger Delta formations.

**Table 2:** Experimental Results from the triaxial experiments.

Sample	Peak Strength	(δ_1_ -δ_3_) max	E @ 5Mpa (MPa)	R^2^
UDCT	80.507303	70.507303	39.775	0.83
UDCB	56.737692	46.737692	23.966	0.85
DOCB	65.782048	55.782048	25.12	0.87
DOCT	51.15778	41.15778	19.091	0.81
DCB	54.684808	44.684808	35.821	0.81
DCT	48.846901	38.846901	18.54	0.76

The confinement pressure that was applied in the study was considered sufficient for the integrity assessment of caprocks for applications such as carbon capture and storage. The stress-strain curves reported are a base for assessing effects of carbon injection. Rutqvist [4] highlighted that any changes in temperature and pressure alters the stress-strain relationships of the reservoir and the caprock hence the need to monitor the initial relationships and the subsequent relationships after the injection. The ranges of the mechanical strength of the caprocks indicated a potential to withstand injection pressure as capillary entrance pressure for carbon dioxide is normally lower than that for methane and can range from less than 0.1–10 MPa, according to Rutqvist [4]. Fracturing of the Niger Delta caprocks due to overpressure is highly unlikely.

## 5 Discussion

The morphological structure that was observed supports that the Niger Delta is favourable for stratigraphical and structural carbon storage. The phases obtained by the powder diffractometer point towards a positive potential for carbon capture and storage. The dominant quartz does not react with carbon dioxide which makes the caprocks resistant to significant geochemical alteration. Garnet, a silicate, behaves similarly to quartz. The minerals could influence the stratigraphical trapping of carbon dioxide in the Niger Delta. The characteristically low permeability of the silicate shale caprocks makes them candidates for stratigraphic trapping as attested by Hameli et al. [8]. Illite clay could play a role in the carbonation of the carbon dioxide that will ensure a permanent storage of the anthropogenic gases in the form of new secondary minerals.

The triaxial results found are comparable to the results obtained by Naderloo [53]. The first non-linear curves could be explained by Hertz pressure phenomena and the closure of the pore spaces inside the core samples as explained by Aadony and Looyeh [54]. The imperfections of the rock particles could have affected the distribution of the forces. The phenomena of Hertz pressure are affected by the roughness of the surfaces in the contact. The triaxial experiments were important to ascertain the compaction of the reservoirs for carbon capture and storage. The caprock's entire mechanical behaviour is influenced by Hertz pressure. The fairly linear section was attributed to a tight compaction after the closure of pores due to compression.

The concave shape that is experienced towards the near the peak strength is also explained in terms of Hertz pressure. The concave shape is attributed to the cracks starting to occur in the core samples, especially in areas with significant stress concentrations. Similar observations were noticed in a research by Naderloo [53].

Hertz pressure has ramifications for the mechanical integrity of depleted reservoirs, rock-fluid interactions, and numerous geo-mechanical processes in the subsurface. It is crucial to comprehend how rocks respond to applied stresses and loads in geological formations.

## 6 Conclusion

The goal of this research was to look at the geo-mechanical and morphological characteristics of caprocks to determine their viability for carbon capture and storage. Our findings indicate that.

• The mineralogical content of the caprocks has the potential suitability to confine carbon dioxide. The quartz and the Garnet are suitable for the stratigraphical trapping of carbon dioxide as they are not reactive.
• The ratio of the minerals indicated that the caprocks in the Niger Delta region are silicate shale. The morphological structure viewed by the SEM showed that the cap rocks are of a packed granular structure which is suitable for inhibiting the buoyant migration of carbon dioxide.
• The triaxial experiments showed that the geo-mechanical strength of the sealing caprocks is within the acceptable and recommended ranges reported in various literature. The silicate shale caprocks for the three depo-belts are classified as strong rocks having a range of peak strength from 48.85 to 80.50 MPa.
• The quasi-elastic behaviour of silicate shale caprocks observed in the experiments makes them good candidates for carbon capture and storage.

Finally, we conclude that our findings gave a satisfactory cap rock strength description suitable for carbon capture and storage. The significance of geo-mechanical processes related to geological carbon dioxide storage cannot be overstated. Overall findings indicated that the depleted oil and gas reservoir systems in the Niger Delta are robust enough for carbon capture and storage, however, rock strength decreases with exposure to supercritical carbon dioxide. It is recommended to repeat the tests after the caprocks are exposed to carbon dioxide.

Further modelling work may be done to assess the feasibility of the cap rock's ability to keep the carbon dioxide from escaping back into the atmosphere and or diffusing into other lithologies. We also recommend that the mineralogical results be used to create geochemical reaction models for geological time reactions. For future work the relationship between rock strength and particular factors such as grain size, sorting, grain shape, and degree and type of diagenetic may be explored.

## Data availability

Data has not been deposited into any publicly available repository. Data will be made available on request.

## CRediT authorship contribution statement

**Itai Mutadza:** Writing – review & editing, Writing – original draft, Visualization, Validation, Software, Resources, Project administration, Methodology, Investigation, Formal analysis, Data curation. **Sunday Sunday Ikiensikimama:** Writing – review & editing, Validation, Supervision, Data curation, Conceptualization. **Ogbonna Friday Joel:** Writing – review & editing, Validation, Supervision.

## Declaration of competing interest

The authors declare that they have no known competing financial interests or personal relationships that could have appeared to influence the work reported in this paper.
